# Micro CT visualization of silver nanoparticles in the middle and inner ear of rat and transportation pathway after transtympanic injection

**DOI:** 10.1186/s12951-015-0065-9

**Published:** 2015-01-27

**Authors:** Jing Zou, Markus Hannula, Superb Misra, Hao Feng, Roberto Hanoi Labrador, Antti S Aula, Jari Hyttinen, Ilmari Pyykkö

**Affiliations:** Hearing and Balance Research Unit, Field of Oto-laryngology, School of Medicine, University of Tampere, Medisiinarinkatu 3, 33520 Tampere, Finland; BioMediTech and Department of Electronics and Communications Engineering, Tampere University of Technology, Tampere, Finland; School of Geography, Earth and Environmental Sciences, University of Birmingham, Birmingham, UK; Nanologica AB, Stockholm, Sweden; Department of Otolaryngology-Head and Neck Surgery, Center for Otolaryngology-Head & Neck Surgery of Chinese PLA, Changhai Hospital, Second Military Medical University, Shanghai, China; Department of Medical Physics, Imaging Centre, Tampere University Hospital, Tampere, Finland; Materials Science and Engineering, Indian Institute of Technology-Gandhinagar, Ahmedabad, India

**Keywords:** Silver nanoparticles, Micro CT, Ear, Animal, Pathway

## Abstract

**Background:**

Silver nanoparticles (Ag NPs) displayed strong activities in anti-bacterial, anti-viral, and anti-fungal studies and were reportedly efficient in treating otitis media. Information on distribution of AgNPs in different compartments of the ear is lacking.

**Objective:**

To detect distribution of Ag NPs in the middle and inner ear and transportation pathways after transtympanic injection.

**Methods:**

Contrast effect of Ag NPs in the micro CT imaging was assessed in a phantom. AgNPs at various concentrations (1.85 mM, 37.1 mM, and 370.7 mM) were administered to rat middle ear using transtympanic injection and cadaver heads were imaged using micro CT at several time points.

**Results:**

The lowest concentration of Ag NPs that could be visualized using micro CT was 37.1 mM. No difference was observed between the solvents, deionized H_2_O and saline. Ag NPs at 37.1 mM were visible in the middle ear on 7 d post-administration. Ag NPs at 370.7 mM generated signals in the middle ear, ossicular chain, round window membrane, oval window, scala tympani, and Eustachian tube for both 4 h and 24 h time points. A gradient distribution of Ag NPs from the middle ear to the inner ear was detected. The pathways for Ag NPs to be transported from the middle ear into the inner ear are round and oval windows.

**Conclusion:**

This study provided the imaging evidence that Ag NPs are able to access the inner ear in a dose-dependent manner after intratympanic administration, which is relevant to design the delivery concentration in the future clinic application in order to avoid adverse inner ear effect.

## Introduction

Silver nanoparticles (Ag NPs) displayed strong activities in anti-bacterial, anti-viral, and anti-fungal studies attributed to the mechanisms of inhibiting the formation of biofilm and destroying viral structures and boosting innate immune response among others [1-5]. Study performed by Radzig et al. supports the hypothesis that Ag NPs exert the antibacterial action through inducing generation of reactive oxygen species and causing DNA damage by oxidative stress, which can be also involved in the mechanisms of antiviral and antifungal activities [6]. Ag NPs also showed excellent behavior in surface-enhanced Raman scattering for the advanced Raman spectroscopy, which has potential for broad range of applications in clinical molecular imaging [7].

Potentially, Ag NPs will be used to treat otitis media and the consequential sensorineural hearing loss through intratympanic administration. Chronic otitis media, characterized by recurrent infections causing pain and purulent otorrhea, is still a significant public health problem affecting 0.5–30% of any given population in developing and developed countries. Complications with sensorineural hearing loss and vestibular impairment were repeatedly reported in the literatures [8-12]. Endolymphatic hydrops secondary to the middle ear infection was demonstrated in both animal model and patient with Meniere’s disease using gadolinium enhancement magnetic resonance imaging (MRI) [13,14]. However, antibiotic is not always efficient because of the appearance of multidrug resistant strains of bacteria. Formation of biofilm was recently reported in the middle ear of patients with chronic otitis media all over the world [15-18]. Through a completely different mechanism, Ag NPs may overcome all the disadvantages of any antibiotics and eliminate the microorganisms with high efficacy in the ear therapy. This therapeutic strategy was encouraged by a clinical study on treatment of relapses of chronic suppurative otitis media using a preparation containing Ag NPs. The study showed that Ag NPs eliminated clinical symptoms and positive dynamics of the objective signs of the disease, such as reduction or termination of pathological exudation and stimulation of the epidermization processes, which was stable during the observation time of 6 months [19]. In order to persuade this novel therapy with sophisticated design, detailed information on distribution and pathway of Ag NPs in the middle and inner ear is necessary but currently lacking in the literature.

Micro computed tomography (CT) has been engaged in middle and inner ear imaging of animals and implicated to be a useful tool to trace kinetics of drugs in the inner ear [20,21]. The gray levels in a CT slice image correspond to X-ray attenuation, which reflects the proportion of X-rays scattered or absorbed as they pass through each voxel, and is affected by the density and composition of the material being imaged. Hence, Ag NPs are speculated to attenuate the X-rays and be visible in micro CT images. In the present work, first a phantom study was performed to check the dose response of the imaging system. Next, an *in vivo* experiment was carried out in rats by injecting Ag NP suspensions with different concentrations into the middle ear cavity and following the kinetics of Ag NPs in the middle and inner ear up to 7 d.

## Results

### Characterization of Ag NPs and potential interaction with artificial perilymph

**Figure 1:**
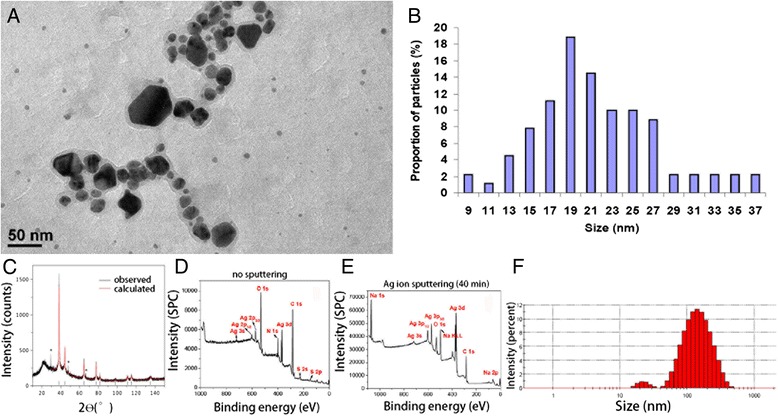
**Characterization result of PVP coated Ag NPs using various analytical techniques. A)** Transmission electron microscopy (TEM) image of NPs showing the polydispersity in size and shape of the PVP coated AgNPs. **B)** TEM particle size distribution of NPs (n = 200, mean = 21 ± 8 nm), **C)** X-ray diffraction pattern for Ag NPs indicating the presence of metallic silver (ICDD 004–0783). **D-E)** XPS analysis on Ag NPs without any sputtering indicating the presence of high amount of organic impurities (PVP used as a surfactant). Sputtered spectrum **(E)** confirms the presence of the organic components only on the surface. **F)** Hydrodynamic size of the NPs when suspended in deionized water, measured using dynamic light scattering.

**Figure 2:**
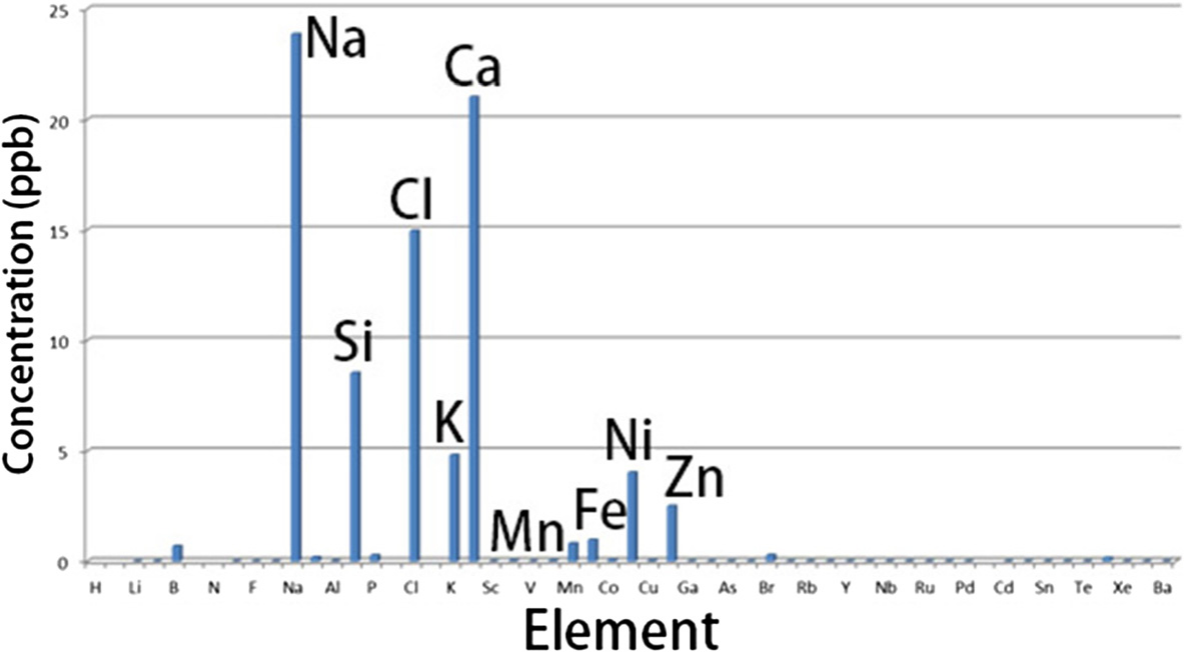
**Level of impurities found in the Ag NPs shown by inductively couple plasma-mass spectrometry.**

The Ag NPs used in this study were highly faceted with a mean size of 21 ± 8 nm. The particles were polydispersed in size and shape, as shown in Figure 1. The transmission electron microscope (TEM) images and size distribution of the particles are shown in Figure 1a. X-ray diffraction (XRD) analysis confirmed the crystalline nature of the particles (ICDD: 004–0783). The mean hydrodynamic size of the particles when suspended in deionized water was 117 ± 24 nm, and the zeta potential was measured to be −20 ± 9 mV. Inductively coupled plasma measurements on the particles showed a very low level of species other than silver, which were mostly cations (Figure 2). Because the nanoparticles were stabilized in the suspension using polyvinylpyrrolidone (PVP), XPS analysis was performed to characterize the surface of the particles. The un-sputtered spectrum of the particles showed a high presence of organic carbon, which was evidently due to the presence of PVP used as the capping agent/surfactant. However, after increasing the sputtering time, the Ag 3 d peak started to appear stronger, suggesting a core shell structure wherein the core was metallic silver and the shell was composed of an organic coating with PVP. Incubation with artificial perilymph for 4 h did not significantly affect the size distribution of the Ag NPs (Table 1).

**Table 1:** **Size distribution of AgNPs in artificial perilymph for 4 h at different dilutions**

**Concentration (dilution)**	**Z**_**mean**_ **(nm)**
x10	106.9 ± 0.3
X100	102.9 ± 0.7
X1000	100.2 ± 1.0
X10000	100.7 ± 1.2

### Sensitivity of micro CT imaging of Ag NPs

**Figure 3:**
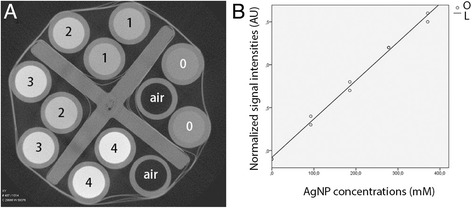
**Sensitivity and linear correlation between signal intensity and Ag NP concentrations shown by micro CT phantom.** Ag NPs were dissolved in H_2_O at variable concentrations (mM) and imaged using micro CT **(A)**. The signal intensities of each dot were normalized by dividing with that of the air and linear correlation with the Ag NP concentrations was estimated **(B)**. Concentrations in A: 0 = H_2_O; 1=, 92.7 mM; 3 = 185.4.4 mM; 3 = 278.0 mM; 4 = 370.7 mM. AU: arbitrary unit; L: linear; O: observed.

The current setup of micro CT showed a detection limit for Ag NPs at a concentration of 37 mM. Good linearity between the signal intensity and Ag NPs concentration was obtained in the range of 37–370.7 mM that were dissolved in H_2_O (Figure 3). Significant correlation was observed between signal intensities of Ag NPs generated in H_2_O and NaCl solutions, but the H_2_O provided significantly higher signal intensities than the NaCl with normalized value of 1.04 (p < 0.001, paired samples t-test).

### Distribution of AgNPs in the middle and inner ear and pathways

**Figure 4:**
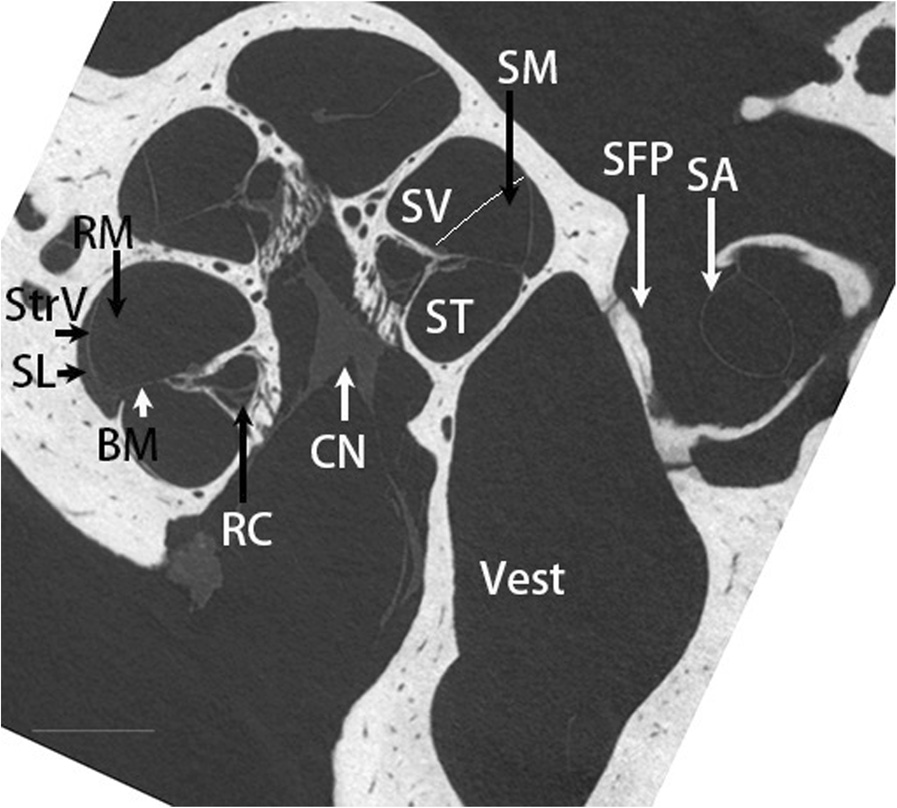
**The heterogeneous fine structures of rat inner ear were demonstrated using iodine-contrasted micro CT.** BM: basilar membrane; CN: cochlear nerve; RM: Reissner’s membrane; SA: stapedial artery; SFP: stapes footplate; ST: scala tympani; SV: scala vestibuli; Vest: vestibule. scale bar = 500 μm.

**Figure 5:**
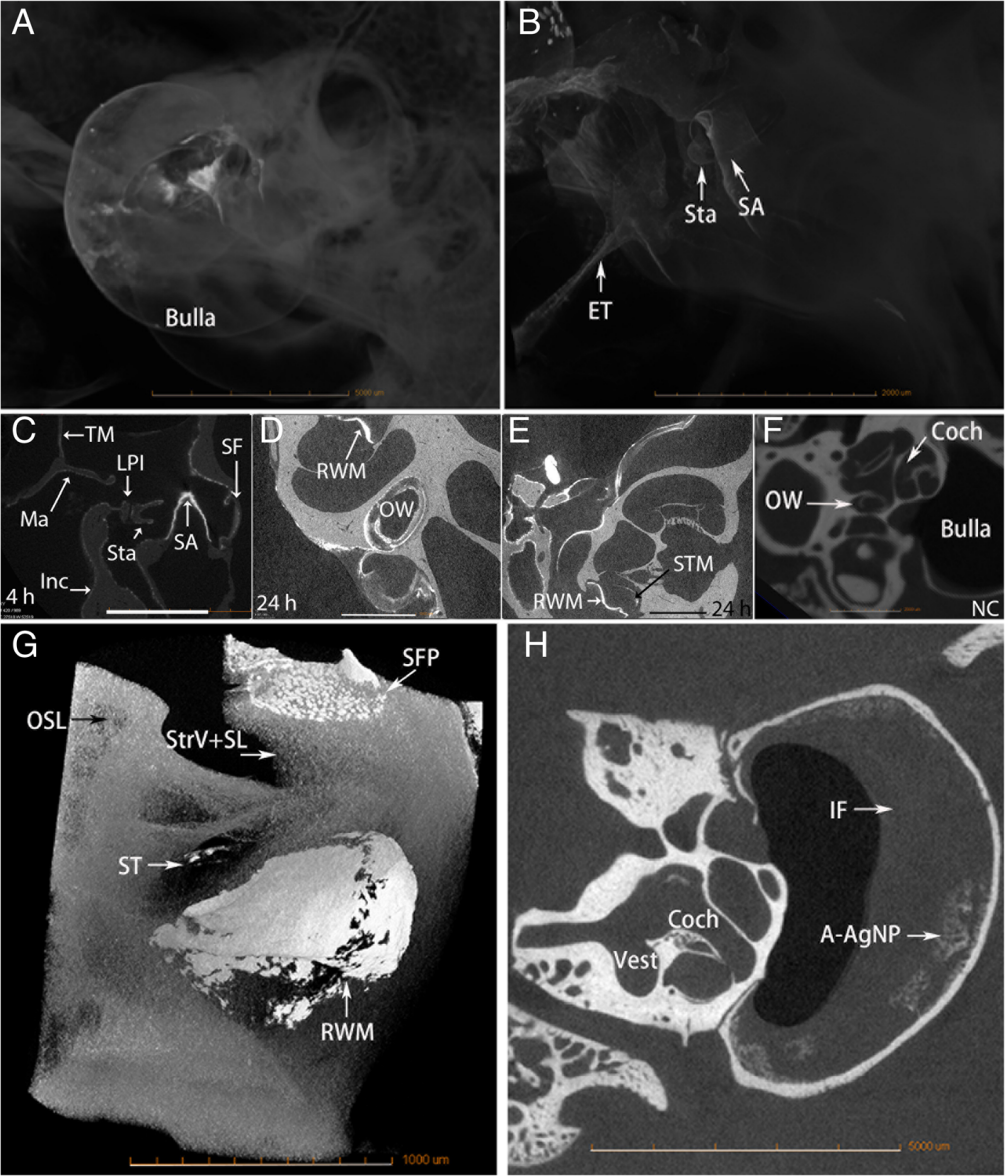
**Distribution of Ag NPs in the ear after transtympanic injection shown by micro CT.** Either 370.7 mM **(A-E, G)** or 37.1 mM (H) of Ag NPs were injected at a volume of 50 μl. At 4 h post-administration (370.7 mM), AgNPs generated bright signal that appeared in the bulla, tympanic membrane (TM) Eustachian tube (ET), and the ossicular chain including malleus (Ma), incus (Inc) and stapes (Sta) **(A-C)**. Abundant Ag NPs were found in the stapedial artery (SA) **(C)**. At 24 h (370.7 mM), abundant distribution of AgNPs was detected in the round window membrane (RWM), oval window (OW), and scala tympani medial wall (STM) of the cochlea **(D, E, G)**. On 7 d (37 mM), middle ear infiltration (IF) and AgNP aggregation (A-AgNPs) were observed (H). No Ag NPs were detected in the ear of non-treatment control (NC) **(F)**. Coch: cochlea; LPI: lenticular process of incus; SF: stapes footplate; ST: scala tympani. Scale bars = 5 mm **(A)**, 2 mm **(B, F)**, 1 mm **(C-E). A-F**, **H**: 4x, Pixel size 21.8498; G: 10x, pixel size 1.7 um.

The heterogeneous fine structures of rat cochlea were demonstrated by iodine-contrast micro CT in Figure 4. The optimized protocol for rat ear micro CT imaging had a resolution of 21.9 μm, which can utilize both the middle ear and inner ear for detecting the distribution of the Ag NPs in both compartments. At 4 h after transtympanic injection of 370.7 mM Ag NPs, the nanoparticles distributed along the middle ear mucosa, diffused to the Eustachian tube, and the extra Ag NPs flowed out into the external ear canal. Abundant Ag NP accumulation on the surface of ossicular chain and stapes artery was detected. The Ag NPs significantly distributed in the round window membrane and continuously moved to the mesothelium of the scala tympani and the annular ligament across the stapediovestibular joint, which is the junctional site between the middle ear and vestibule (Figure 5). At 24 h, Ag NPs showed abundant distribution on in the round window membrane and oval window, and became more visible within the cochlea (Figure 5). Ag NPs was detected in the middle ear mucosa at 4 h post-transtympanic injection at 37 mM in one rat. Aggregated Ag NPs were visualized in both middle ear and cochlea on 7 d after injection at 37 mM (Figure 5). Higher estimated concentrations of Ag NPs in various locations of the ear than the applied concentrations supported the aggregation or accumulation of Ag NPs in the corresponding area (Table 2). However, transtympanic injection of Ag NPs at 1.85 mM did not produce any signal of Ag NP at the time points of 4 h, 24 h, and 7 d post-administration. There was not any fluid detected in the middle ear cavity at these time points indicating that there was no infiltration.

**Table 2:** **Concentrations (mM) of AgNPs distributes in various locations of rat ear after transtympanic injection measured by μCT**

**AgNP con delivered**	**Time**	**ME**	**ME-flu**	**Mall**	**Inc**	**Stap**	**StapArt**	**StapFoot**	**OW**	**RWM**	**Coc**	**EEC**
371	5 h	1270		547	677	500	769		1038		639	1177
371	4 h	1084		232	269	408	677		232			
371	24 h			816	639	769		639	1177			
371	24 h			677	593	723	1084	769	955	1177	593	
37	4 h	139										
37	1 w	232	93									
37	1 w	185										

Intensities in various locations of rat ear after transtympanic injection of AgNPs were normalized by the intensities of the cochlear perilymph imaged by μCT. The concentrations of AgNPs were estimated using the formula of y = 4.88x-4.86 obtained in a phantom study, where “y” is the concentration and “x” is the normalized intensity. AgNP con: AgNP concentration; Coc: cochlea; EEC: external ear canal; Inc: incus; ME: middle ear mocusa; ME-flu: middle ear fluid; Mall: malleus; OW: oval window; RWM: round window membrane; Stap: stapes; StapArt: stapedial artery.

## Discussion

The present work demonstrated that the PVP-coated Ag NPs were visible in the ear by micro CT after transtympanic injection and entered in the inner ear through the round and oval windows. The detected bright signals in the ear by micro CT could be either aggregated Ag NPs or silver compound formed upon contacting the extracellular or cellular fluids. Ag NPs encounter different extracellular environments in the external ear canal, middle ear, and inner ear. The plentiful perilymph in the inner ear may interact with Ag NPs and form a compound immediately after the entry. However, the incubation of Ag NPs with artificial perilymph did not change the size distribution over a period of 25 h, suggesting that the bright signals in the ear represent the Ag NPs. It was reported that silver might be developed as a radiographic contrast agent in dual-energy breast X-ray imaging [22]. However, the detection sensitivity of Ag NPs by micro CT is rather low and the detection limit is 37 mM, a concentration that demonstrated toxicity in the rat ear [23]. These results did not support that the current form of Ag NPs will be used as a contrast agent for CT imaging. Clinical feasibility, however, warrants further studies.

The oval window pathway was recently proved to be more efficient than the round window to transport chelated-gadolinium from the middle ear to the inner ear in animals and human shown by MRI [24,25]. The pathways for the Ag NPs to enter the inner ear were clearly shown to be the round and oval windows. This indicates that the oval window potentially has a broad spectrum of substance transportation in addition to chelated-gadolinium. At 24 h post-administration to the middle ear at a concentration of 370.7 mM, Ag NPs accumulated in the round window membrane and oval window, and concentrated in the scala tympani, which indicates that the entry of Ag NPs into the inner ear is a dynamic process. This conclusion was further supported by the quantification of Ag NPs in various regions of the ear (Table 2). Obvious Ag NP signal was detected in the middle ear after administration at a concentration of 37.1 mM that was the lowest detection limit of the present setup, which may result from accumulation or aggregation of Ag NPs in the middle ear as supported by the quantification result (Table 2). No signal was detected in the inner ear when Ag NPs were administered at a concentration of 37 mM. This might be caused by the low sensitivity of micro CT visualization. Our explanation is that the layer of Ag NPs formed on tissue surfaces of the inner ear is too thin to raise the value of the voxel as a result of the partial volume effect (the grayscale value of a voxel is the volume fraction weighted sum of all the materials present in the voxel). A previous study demonstrated that hearing loss occurred in rats after middle ear administration of 37.1 mM Ag NPs, which suggests that certain amount of Ag NPs (below the detection threshold of the micro CT) should have entered the inner ear [23].

The long term remaining of Ag NPs in the middle ear cavity for 7 d post-transtympanic injection supports that Ag NP is a potential candidate to combat otitis media. Although no signal was detected in the inner ear on 7 d post-administration of 37.1 mM Ag NP, it did not rule out the penetration of Ag NPs into the inner ear because hearing loss and pathological changes were detected in rats exposed to Ag NPs at this concentration [23]. 1.85 mM Ag NPs did not generate either micro CT signal of AgNPs or infiltration in the middle ear cavity. No infiltration indicates that 1.85 mM of Ag NPs is a safe level for the ear, which is in accordance with our observation that neither hearing loss nor cytokine up-regulation in the inner ear was induced by Ag NPs at this concentration (unpublished data). Importantly, 1.85 mM of Ag NPs is sufficient to inhibit biofilm formation during bacterial infection which only demands 0.1-2 mM Ag NPs [26].

In addition, the extra Ag NPs were secreted to the nasal pharynx through the Eustachian tube and flowed to the external ear canal through the tympanic membrane penetration. Dysfunction of the Eustachian tube is a common complication of otitis media. The distribution of Ag NPs in the Eustachian tube suggest that Ag NPs may have direct effect on the extension of otitis media. The dendrimer-stabilized silver nanoparticles, that have similar sizes as the Ag NPs utilized in the present study, were reportedly effective in X-ray computed tomography (CT) imaging and stable in water, PBS buffer, fetal bovine serum, and resistant to changes in pH and temperature [27]. There is a possibility that the dendrimer-stabilized silver nanoparticles may be used as a contrast agent in the CT imaging of the external, middle, and inner ears and the Eustachian tube in the future based on the present results.

## Conclusions

The distribution of Ag NPs in the middle and inner ear is visible by micro CT and a gradient concentration from the middle ear to the inner ear was detected. The pathways for Ag NPs to be transported from the middle ear into the inner ear are round and oval windows. This study provided the imaging evidence that Ag NPs are able to access various regions of the ear after intratympanic administration in a dosage-dependent manner, which is relevant to design the delivery concentration in the future clinic application in order to avoid adverse inner ear effect.

## Materials and methods

### Materials

The Ag NPs was supplied by Colorobbia (Firenze, Italy). Ten male Sprague Dawley rats, weighing between 330 g and 410 g, were maintained in the Experimental Animal Unit, School of Medicine, University of Tampere, Finland. All animal experiments were approved by the Ethical Committee of University of Tampere (permission: ESAVI/3033/04.10.03/2011). Animal care and experimental procedures were conducted in accordance with European legislation. Two rats were assigned into each group with respect to concentrations of AgNPs and imaging time (Table 3). Animal care and experimental procedures were conducted in accordance with European legislation. All experiments were performed under general anesthesia with intraperitoneal injection of a mixture of 0.8 mg/kg of medetomidine hydrochloride (Domitor, Orion, Espoo, Finland) and 80 mg/kg of ketamine hydrochloride (Ketalar; Pfizer, Helsinki, Finland) followed by intramuscular injection of Enrofloxacin (Baytril®vet, Orion, Turku, Finland) at a dose of 10 mg/kg to prevent potential infection. During experiments, the animal’s eyes were protected by Viscotears® (Novartis Healthcare A/S, Denmark).

**Table 3:** **Assignments of rats in micro CT measurements and distribution of AgNPs in the ear post-intratympanic administration**

**AgNPs conc**	**370.7 mM**	**37.1 mM**	**1.85 mM**		
**Time points**	4 h*	24 h*	4 h*	7 d2*	7 d*
Locations of AgNPs in the ear	ME, OC, SA, RWM, OW, ET, ST	ME, OC, SA, RWM, OW, ET, ST	ND	ME	ND

*Two rats were assigned into each group. conc: concentration; ET: Eustachian tube; ME: middle ear; ND: not detected; OC: ossicular chain; OW: oval window; RWM: round window membrane; SA: stapes artery; ST: scala tympani.

### Characterization of Ag NPs

The Ag NPs were dispersed in water (370.7 mM) and characterized using a range of analytical techniques, to assess various physicochemical properties (eg. size, shape, zeta potential, surface properties etc.). For TEM measurements, a diluted suspension of Ag NPs was deposited on a copper grid for TEM imaging (Hitachi 7100, 100 kV). XRD was performed on the NPs using an Enraf-Nonius diffractometer coupled to INEL CPS 120 position-sensitive detector with Co-K_α_ radiation, and the phase identification was performed using STOE software. The hydrodynamic size and zeta potential of the nanoparticles were measured using a Malvern Zetasizer (Malvern Instruments, Malvern, UK). ICP-AES (Varian Instruments) analysis was performed to determine the initial concentration of silver in the aqueous nanoparticulate suspension and to measure the level of any impurities present in the matrix. X-ray photoelectron spectroscopy (XPS, Omicron Nanotechnology) was used to study the chemical composition and chemical state of the Ag NPs. The XPS analyses were performed in an ultra-high vacuum medium (pressure of 10^−10^ mbar) using an Al, Kα (hν = 1486.7 eV) X-ray source, with power given by the emission of 16 mA at a voltage of 12.5 kV. For the silver element, the high-resolution spectra were obtained with analyzer pass energy of 50 eV and a step size of 0.01 eV. The argon ion flux was employed to sputter the surface and remove the adsorbed species, with an energy of 3.5 kV, emission of 20 mA, and incidence angle of 45° over a period of 20 and 40 min. The binding energies were referred to the carbon 1 s level, which was set as 284.6 eV.

### Potential impact of perilymph on Ag NPs

Since the Ag NPs will interact with perilymph once enter the inner ear, the potential impact of perilymph on Ag NPs was evaluated. The artificial perilymph containing 145.5 mM NaCl, 2.7 mM KCl, 2.0 mM MgSO_4_, 1.2 mM CaCl_2_, and 5.0 mM HEPES, with the pH adjusted to 7.4, was prepared as previously reported [28]. Ag NPs were diluted with artificial perilymph at 10, 100, 1000 and 10000-fold and stored at room temperature for 4 h before the size distribution was measured using DLS (Malvern Zeta Sizer Nano ZS, UK). For the change in the DLS over 25 h, the dilutions were 10-fold.

### Micro CT studies

#### Phantom study

The first round experiment was designed to check the sensitivity of the imaging system using solutions of Ag NPs with broad concentration range (370.7 mM, 37.1 mM, 3.7 mM, 0.37 mM, and 0.037 mM) that were prepared with either deionized H_2_O or saline and placed into plastic phantom tubes arranged concentrically on the modified piston rod of a 50 ml syringe. Negative controls were prepared using saline. Each sample was prepared in duplicate. The phantom was firmly installed on the specimen stage of the MicroXCT-400 (Carl Zeiss X-ray Microscopy, Inc, Jena, Germany) and imaged using the following parameters: Voltage 120 kV, current 83 μA, pixel size 33.95 μm, exposure time 0.5 s. The detection limit of the imaging system with the defined parameters was shown to be 37.1 mM based on the first round experiment. The second round experiment was performed using solutions of Ag NPs with smaller concentration range (370.7 mM, 278.0 mM, 185.4 mM, 92.7 mM) suspended in H_2_O according to the above protocol to determine the accurate correlation between the concentration and signal intensity.

#### Animal study

Under general anesthesia, 50 μl of Ag NPs at defined concentrations were injected into the left middle ear cavity through the tympanic membrane penetration under an operating microscope according to a previously reported procedure [29]. After injection, the animals were kept in the lateral position with the injected ear oriented upward for 15 min to ensure the sufficient amount of Ag NPs to remain in the middle ear cavity before intraperitoneal injection of Antisendan (atipamezole hydrochloride, Orion Pharma, Finland) (2 mg/kg) to accelerate recovery from anesthesia. At certain observation time points post-administration (Table 3), animals were injected intraperitoneally with pentobarbital sodium at a dosage of 100 mg/kg. The temporal bones were fixed through cardiac perfusion with 0.01 M PBS containing 0.6% (v/v) heparin (pH 7.4) and then 4% paraformaldehyde (Merck, Espoo, Finland).After decapitation, the animal head was further fixed with 4% paraformaldehyde for 2 h, covered with parafilm, and placed on the specimen stage of the micro CT. During imaging, three objectives were used, 1X for the large field of view images, 4X for the images that were focused onto the cochlea, 10x for imaging the oval and round windows. The voltage varied from 60 to 120 kV, the source distance was adjusted to 60–100 mm, and the detector distance was 38–40 mm. The pixel size ranged from 1.7 to 35.4 μm according to different setup parameters. Afterwards, one bulla was processed for iodine-contrast micro CT imaging in order to demonstrate the soft tissue in the inner ear. The stapes was displaced and about 5 μl iodixanol (VisipaqueTM, 320 g I/ml, GE Healthcare, Helsinki, Finland) was infused into the inner ear using a high-performance polyimide tubing (MicroLumen, Tampa, FL, USA) that was connected to polyethylene tubing (PE10, Becton, Dickinson and Company, Franklin Lakes, NJ, USA) [28].The images were acquired with a 4x-objective, source voltage of 40 kV and current 200 μA, pixel size of 5.6 μm. Images were collected using the Xradia TXMController software and reconstructed using the Xradia TXMR econstructor software.

### Image analysis and statistics

Signal intensities in the region of interest were evaluated using Image J 1.46r software (National Institutes of Health, Bethesda, MD). Linear equation was used for the curve estimation between Ag NP concentration and signal intensity obtained using micro CT in phantom. Paired samples T-test (IBM SPSS statistics 20) was used to compare the signal intensity generated by Ag NPs in deionized H_2_O and NaCl solutions. Intensities in various locations of rat ear after transtympanic injection of Ag NPs were normalized by the intensities of the cochlear perilymph imaged by μCT. The concentrations of Ag NPs were estimated according to the linear curve obtained in the phantom study.

## Abbreviations

AgNPs: Silver nanoparticles
CT: Computed tomography
MRI: Magnetic resonance imaging
PVP: Polyvinylpyrrolidone
TEM: Transmission electron microscope
XRD: X-ray diffraction
